# Selection for Cheaper Amino Acids Drives Nucleotide Usage at the Start of Translation in Eukaryotic Genes

**DOI:** 10.1016/j.gpb.2021.03.002

**Published:** 2021-03-17

**Authors:** Na L. Gao, Zilong He, Qianhui Zhu, Puzi Jiang, Songnian Hu, Wei-Hua Chen

**Affiliations:** 1 Key Laboratory of Molecular Biophysics of the Ministry of Education, Hubei Key Laboratory of Bioinformatics and Molecular-imaging, Department of Bioinformatics and Systems Biology, College of Life Science and Technology, Huazhong University of Science and Technology, Wuhan 430074, China; 2 Institute for Computer Science and Cluster of Excellence on Plant Sciences, Heinrich Heine University, D-40225 Duesseldorf, Germany; 3 CAS Key Laboratory of Genome Sciences and Information, Beijing Institute of Genomics, Chinese Academy of Sciences, Beijing 100101, China; 4 State Key Laboratory of Microbial Resources, Institute of Microbiology, Chinese Academy of Sciences, Beijing 100101, China; 5 Beijing Advanced Innovation Center for Big Data-Based Precision Medicine, Interdisciplinary Innovation Institute of Medicine and Engineering, Beihang University, Beijing 100191, China; 6 University of Chinese Academy of Sciences, Beijing 100049, China

**Keywords:** Macroevolution, Prioritization of selective forces, Energy efficiency, Transcription, Translation initiation

## Abstract

Coding regions have complex interactions among multiple selective forces, which are manifested as biases in nucleotide composition. Previous studies have revealed a decreasing GC gradient from the 5′-end to 3′-end of coding regions in various organisms. We confirmed that this gradient is universal in eukaryotic genes, but the decrease only starts from the ∼ 25th codon. This trend is mostly found in nonsynonymous (ns) sites at which the GC gradient is universal across the eukaryotic genome. Increased GC contents at ns sites result in cheaper amino acids, indicating a universal selection for **energy efficiency** toward the N-termini of encoded proteins. Within a genome, the decreasing GC gradient is intensified from lowly to highly expressed genes (more and more protein products), further supporting this hypothesis. This reveals a conserved selective constraint for cheaper amino acids at the translation start that drives the increased GC contents at ns sites. Elevated GC contents can facilitate **transcription** but result in a more stable local secondary structure around the start codon and subsequently impede **translation initiation**. Conversely, the GC gradients at four-fold and two-fold synonymous sites vary across species. They could decrease or increase, suggesting different constraints acting at the GC contents of different codon sites in different species. This study reveals that the overall GC contents at the translation start are consequences of complex interactions among several major biological processes that shape the nucleotide sequences, especially efficient energy usage.

## Introduction

GC content is one of the most important aspects of nucleotide sequence composition, and it has been extensively studied [1], [2], [3], [4], [5], [6]. Recently, more details of the functional importance of GC content have been revealed. For example, due to an intrinsic trade-off in the codon table, mRNA templates that consist of more energetically costly nucleotides often encode cheaper amino acids. Since GC are energetically more expensive than AT, GC content has become an important indicator for the relative amounts of cellular resources invested in making nucleotides *versus* amino acids [7], [8]. Increased GC contents at nonsynonymous (ns) sites in the coding region increase the costs of the mRNA template but decrease the costs of encoded amino acids. Selection for amino acids is prioritized because protein synthesis is at the downstream end of the information amplification process [7]. It is unsurprising that selection for the usage of cheaper amino acids in highly expressed genes always results in higher GC contents, especially at ns sites.

Higher GC contents at synonymous sites could increase the mRNA levels of mammalian genes [9]. This effect is not due to different translation rates of GC-rich and GC-poor mRNAs but due to more efficient transcription or mRNA processing [9]. These results are consistent with the fact that GC contents in coding regions are always higher than in intergenic regions of eukaryotes, while transcribed non-coding RNAs have intermediate GC contents. Furthermore, the results also suggest that different codon sites may be constrained by different selective forces, with some being constrained by multiple forces.

Increased GC contents in coding regions are not always beneficial. In both bacteria and eukaryotes, less stable local secondary structures (LSSs) with higher minimal folding energy (MFE) are preferred at the translation start in order to facilitate translation initiation [10], [11], [12], [13], [14], which is the most important speed-limiting step of protein translation [15]. Random sequences with increased GC contents often generate more stable LSSs, because the GC pair is bound by three hydrogen bonds, while the AT/AU pair is bound by only two. Increased GC contents at the translation start may impede efficient translation initiation by forming more stable LSSs. In human cells, mRNAs with less stable LSSs around the translation start have a higher protein/mRNA ratio, suggesting the role of LLSs in translation efficiency. In addition, it is known that eIF4A, an evolutionarily conserved eukaryotic initiation factor-4A family that is required for the binding of mRNA to 40S ribosomal subunits, consists of several helicases that function to unwind double-stranded RNA [16], [17].

Selection for less stable LSSs in bacteria is manifested as decreased local GC contents centering at the start codon [10], consequently resulting in the use of rare codons in bacteria that prefer high GC contents at their synonymous sites (*i.e.*, bacteria with overall GC contents higher than ∼ 45%) and the use of frequent codons in bacteria that prefer low GC contents [10]. Similar results have been observed in eukaryotes [18]. The use of rare codons may lead to a significant increase in decoding time in yeast [19], likely due to lower concentrations of the corresponding tRNAs [20]. Codon usage significantly contributes to translational efficiency. Selection for translational efficiency has contributed significantly to codon usage and has subsequently been manifested as nucleotide usage biases. Translation appears to typically be limited by initiation [21].

Previous studies have identified several selective forces that constrain the sequence composition in the coding region. These include efficiencies for energy usage, transcription or mRNA processing, and translation initiation. One region or even one class of codon sites could be simultaneously constrained by multiple selective forces. It is therefore interesting to study the complex interactions among these constraints in eukaryotes to determine how they shaped the sequences at both nucleotide and protein levels, and how they are prioritized in different eukaryotes.

## Results and discussion

### Universal decreasing GC gradients along the coding region

**Figure 1:**
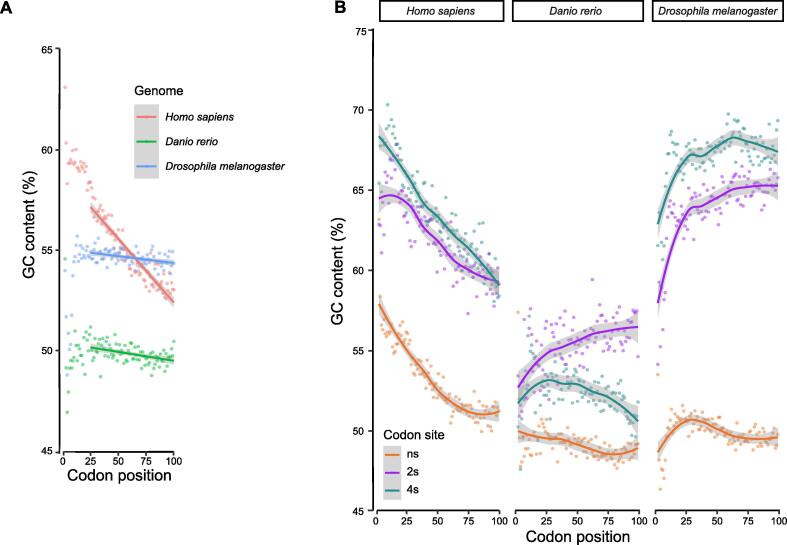
**GC gradients for the first 100 codons of the coding regions in selected species** **A.** Overall GC contents of the first 100 codons for *Homo sapiens* (red), *Danio rerio* (green), and *Drosophila melanogaster* (blue). Each dot represents the average GC content of the codons at the corresponding codon position across all valid genes of a species (see the “Identification of GC gradients in CDSs” section in Materials and methods for details). The linear regression lines were obtained using the 26th–100th codons; the first 25 codons were excluded because they were subjected to constraints for less stable LSSs. **B.** Averaged GC contents of the first 100 codons as a function of codon positions, separated by codon sites. LOESS lines were plotted to better illustrate the general trends. The results for other parts (middle and end) of the coding region are shown in Figure S1. CDS, coding sequence; LSS, local secondary structure; LOESS, locally estimated scatterplot smooth; ns, nonsynonymous; 2 s, two-fold synonymous; 4 s, four-fold synonymous.

**Figure 2:**
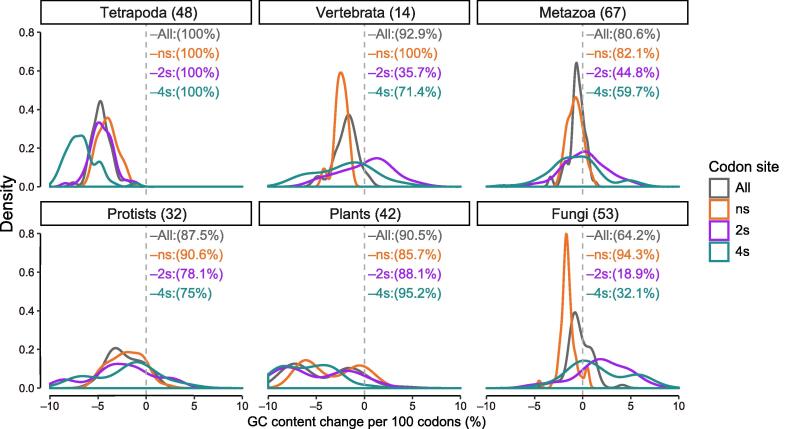
**Decreasing GC gradients at the translation start is universal across eukaryotic species** The X-axis shows the GC content change per 100 codons in the starting part of the coding region that spans 100 codons from the start codon. Species are grouped into broad taxonomic groups. The number of species in each group is indicated in the parenthesis next to the group name. The results for GC gradients in the middle and end parts of the coding region are shown in Figure S2.

In the majority of the 259 eukaryotic genomes studied, we observed a negative GC gradient at the 5′-ends of the coding sequences (CDSs). The GC content was higher at the translation start and then decreased along the direction of translation (Figure 1A, Figure 2). In the selected species shown in Figure 1, this negative gradient is apparent at the first 100 codons and is mostly found at the ns sites, which account for ∼ 60% of the CDS (see Materials and methods). To quantify the extents of the gradients, for each species we applied linear regression to the first 100 codons and obtained slopes for the CDSs, as well as the ns, two-fold synonymous (2 s), and four-fold synonymous (4 s) sites. A slope value indicates the GC content change per 100 codons (%). We removed the first 25 codons from the calculation because they were constrained by various selective forces [10], [18]. As shown in Figure 2, the slopes of the CDSs are negative in the majority of the species studied.

A previous study has suggested that certain signals such as tRNA-adaptation index (tAI) and selection for weak RNA folding could extend beyond the 25th codon [22]. However, for the GC content, it is evident that the first 25 codons are different from the downstream codons, especially on ns sites (Figure 1B). We found that the decreasing GC gradient could be further extended to 150 codons downstream of the translation start (Figure S3).

Some amino acids, including phenylalanine, valine, threonine, tryptophan, methionine, leucine, isoleucine, lysine, and histidine, cannot be synthesized from other substrates and thus must be supplied in the diet. They are often referred as to essential amino acids (data obtained from https://en.wikipedia.org/wiki/Essential_amino_acid, accessed on Oct 6, 2018). We found similar GC gradients at the translation start in both essential and non-essential amino acids (Figure S4), suggesting that essential amino acids are not a unique contributor to our observations.

## Distinct GC gradients at different codon sites across species indicate multiple selective constraints

**Figure 3:**
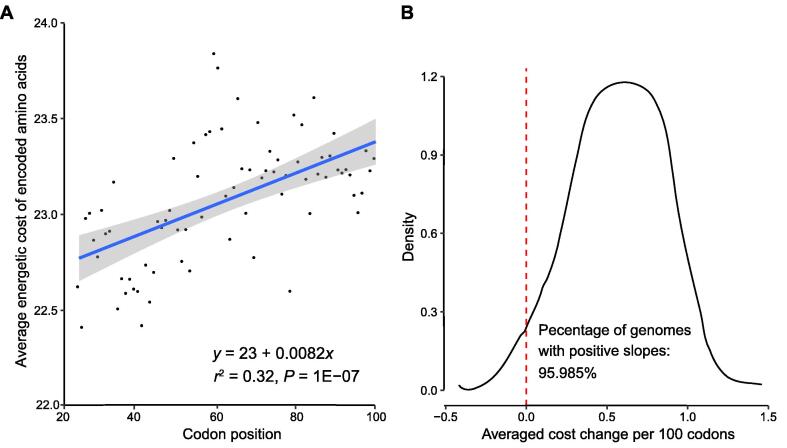
**Energetically cheaper amino acids are used at the N-termini of proteins** **A.** An example of the trend in *Homo sapiens*. Each dot represents the average energetic cost of encoded amino acids at the corresponding codon position (from the 26th codon to the 100th codon) across all valid human genes. The blue line represents LOESS over the dots. Linear regression was applied to the 26th codon to the 100th codon of the starting part to describe the relationship between the energetic costs and codon positions. As indicated in the equation at the bottom-right corner, a positive slope value (0.0082 in the equation) indicates that cheaper amino acids are preferably used toward the start codon, and the costs increase along the direction of translation. **B.** Density plot summarizing the distribution of slope values in the 259 eukaryotic genomes.

By separating codons into codon sites according to their degeneracy, we found distinct patterns of GC contents at these sites in different species. Negative slopes at the ns sites were found in most species. At ns sites, the first parts of the CDSs have the highest GC contents compared with the remainder of the CDSs. G and C are energetically more expensive than A and T/U, respectively, but mRNA templates with high GC contents code for energetically cheaper amino acids [7]. From the perspective of energy efficiency, the use of cheaper amino acids has higher priority than the use of cheaper nucleotides, because protein synthesis is at the end of the information path from DNA to mRNA to protein. This is known as the central dogma of genetics: at each step of the central dogma, the genetic information amplifies and consumes more energy/cellular resources than the previous step. Therefore, higher GC contents at the starting parts of the CDSs may represent an evolutionarily conserved mechanism for energy efficiency. The negative GC gradient from the translation start is thus likely a conserved means to account for premature ribosome drop-offs during translation elongation [23], [24], which should impact the protein N-termini more often than downstream. Consistently, we found that the cheaper amino acids are preferably used at the translation start (Figure 3).

We estimated that for each protein produced in human, an average of ∼ 30 ATPs could be saved in the first 100 codons due to the positive slope (shown in Figure 3A) compared with the 100 codons in the middle. The estimated number of saved ATPs can be calculated using the following equation:

$$\frac{\mathrm{slope}\times n}{2}\times 100$$

where *slope* refers to the slope calculated by applying a linear regression model to the first 26–100 codons and *n* refers to the number of codons used in the linear regression model.

Positively charged amino acids are not randomly distributed around the start codon and the following coding region [25], [26]. These amino acids could cause ribosome pausing and consequently increase ribosome drop-off rates [27]. In addition, proline could also stop peptide bond formation and stalling translation [28], [29]. To test whether the avoidance of those amino acids could explain the negative GC gradient at ns sites, we removed those amino acids from our analysis. Removing the positively charged amino acids and proline did not change our results (Figure S5).

Conversely, GC gradients at 2 s and 4 s sites are often species- and taxon-specific (Figure 2). Depending on the species, GC gradients at these codon sites could show either similar (Figure 1B, right panel) or distinct patterns (Figure 1B, middle panel). These results also indicate that GC-biased gene conversion (gBGC), which is believed to be the underlying mechanism for the GC gradients in angiosperm genomes [30], [31], is insufficient to explain our observations in other species. The gBGC refers to a meiotic repair bias that favors G and C over A and T alleles in high-recombining genomic regions and is believed to be a major contributor to genomic GC evolution [32], [33], [34], [35]. Because the A-to-G and C-to-T changes at 2 s sites often do not change encoded amino acids, the gBGC model predicts similar overall GC content patterns at 2 s and 4 s sites, which is not true in most of the vertebrate species (Figure 2). In addition, there is no evidence for gBGC in *Drosophila melanogaster* [36]. This is consistent with the fact that coding genes on the sex chromosomes in *Drosophila* have higher GC contents than autosomal genes, which contradicts the prediction of gBGC. Overall, our results suggest that, independent of gBGC, there are additional selective forces that increase the GC contents in coding regions, including selection for transcription efficiency that increases with higher GC contents at synonymous sites [9]. These forces are likely to be universal, as the GC contents of coding regions of all eukaryotes are higher than those in intergenic regions. However, species-specific constraints must exist to shape the different patterns at 2 s and 4 s sites in different species (Figure 1, Figure 2).

### GC gradients at ns sites are stronger in genomes with higher coding GC contents

**Figure 4:**
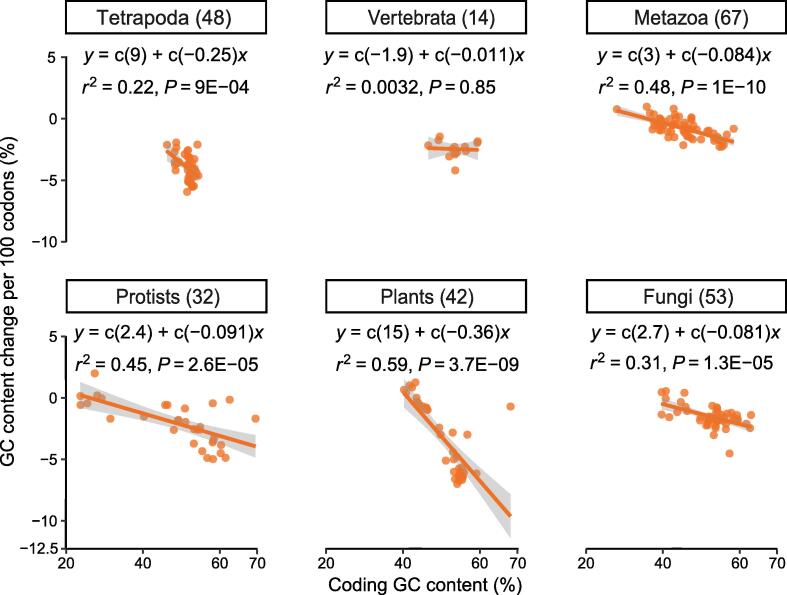
**GC gradients at ns sites of the start codon correlate with the overall coding GC content**Each dot represents a genome. Species are grouped into broad taxonomic groups (Table S1). The number of species in each group is indicated in the parenthesis next to the group name.

We found a significant negative correlation between the slopes of GC gradients at the ns sites and the overall coding GC contents in five out of six broad taxonomic groups (Figure 4). Applying linear regression to these correlations, we found that about 22% (tetrapoda) to 59% (plants) of variation in the slopes of the GC gradients could be explained by the overall coding GC contents. “Vertebrata” was the only exception, possibly due to the fact that few species (14) were available in Ensembl [37] for this group (see Material and methods). Overall, coding GC contents explained ∼ 30% of the variation in the slopes of GC gradients in the 259 species investigated here (Figure S6).

The correlations have two interesting characteristics. First, the slopes of the linear regression (indicated by the solid lines) are different among groups, possibly due to their adaptation to unique environments. Second, the GC content of 40% seems to be the threshold for positive *versus* negative GC gradients. Higher GC contents code for cheaper amino acids; hence, higher overall coding GC content may represent a stronger selective constraint for energy efficiency in the corresponding genomes. Therefore, the higher the overall coding GC content, the higher is the need for energy efficiency and the stronger the negative GC gradient at the translation start.

GC gradients in eukaryotes have been described in plants such as Gramineae species (grasses) [31], angiosperms (flowering plants) [30], and seed plants [38]. Research has focused on inter- and intra-species variations [30], [38]; however, global factors affecting the strengths of GC gradients have not been discussed. We found that at ns, 2 s, and 4 s sites as well as coding regions as a whole, the slopes of the GC gradients at the first 100 codons correlate significantly in eudicot and monocot plants (Figure S7). Overall, coding GC content in a genome could explain 93% of the strength (slopes) of the GC gradients.

### Negative GC gradients are increasingly stronger from metazoan to vertebrata to tetrapoda species

We found increasingly stronger (more negative) GC gradients from metazoan to vertebrata to tetrapoda species. As shown in Figure 2, the peaks of the density plots at ns sites shifted from −1 in the metazoa group (GC content decreased 1% per 100 codons) to −2.5 in the vertebrata group and further to −5 in tetrapoda. The 4 s sites and the overall CDSs showed similar trends, but the 2 s sites did not.

The mRNAs with higher GC contents at the translation start may form more stable secondary structures. This would consequently impede translation initiation, which is the most important speed-limiting step of translation. A less stable secondary structure around the translation start is preferred by bacteria [10], [11] as well as human [12]. For example, in human, the translation initiation efficiency, as indicated by the protein/mRNA abundance ratio, correlates significantly with the MFE of the sequence in a 39-bp window near the start codon [12]. More importantly, the less stable the translation start compared to the downstream coding region, the higher the translation initiation efficiency [12]. These results are consistent with our observations that despite the trend of higher GC contents toward the translation start, up to 25 codons after the start codon often showed lower GC contents, although the decrease was weakened in metazoan compared to vertebrata and tetrapoda species (Figure 1).

**Figure 5:**
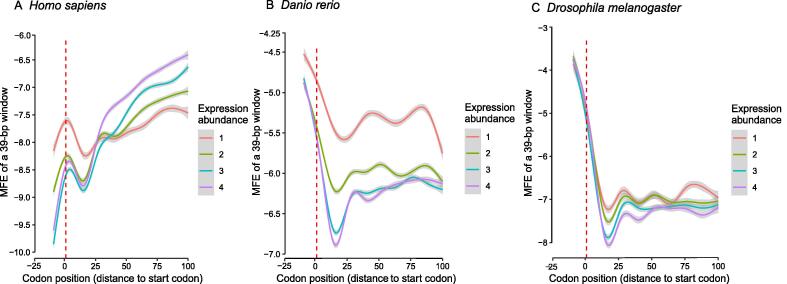
**Highly expressed genes and genes in higher species tend to form more stable LSSs at the translation start** Shown here are the results for selected model organisms, including *Homo sapiens* (**A**), *Danio rerio* (**B**), and *Drosophila melanogaster* (**C**). The MFE values were calculated using a 39-bp window, with a codon at the center, plus 18-bp flanking on each side. This window moved along CDSs with a step of 3 bp; it may extend to the 5′-UTR regions. Genes were grouped into equal-sized bins according to their expression abundances in normal tissues: 1 to 4, from lowest to highest. MFE, minimal folding energy.

Stronger GC gradients and higher GC contents at the translation start often mean more stable LSSs for the transcribed mRNAs. We found that the LSSs around the start codon, as measured by MEF, were increasingly stronger from *D. melanogaster* to *Danio rerio* to *Homo sapiens* (Figure 5). For example, in *D. melanogaster*, the LSSs around the start codon were less stable as compared with downstream coding regions (Figure 5C). In *D. rerio*, the differences still existed but were less significant (Figure 5B). In *H. sapiens* (Figure 5A) as well as in the mouse (Figure S8), the LSSs around the start codon were more stable than the immediate downstream coding regions (regions from about the 10th codon to the 20th codon). These results indicate that the two selective constraints that shape the coding MEFs, namely the selection efficiencies for translation initiation and GC content, are prioritized in different species with the former being deprioritized in higher animals.

More highly expressed genes often have higher GC contents, likely due to the fact that: 1) higher GC contents at ns sites correspond to cheaper amino acids, and 2) higher GC contents at synonymous sites correspond to higher transcription efficiency in eukaryotes [9]. It is possible that highly expressed genes tend to have more stable LSSs around the start codons. In selected species within different taxonomic groups, this appears to be the case and this trend is more obvious in higher animals such as *D. rerio* and *H. sapiens* (Figure 5).

## Conclusion

We describe a universal trend of elevated GC content toward the start codon that manifests as a decreasing GC gradient from the 5′-end to the 3′-end of the coding region. This trend is mostly contributed by the ns sites, which consist of ∼ 60% of all codon sites. The GC gradients at the ns sites are universal across most of the 259 eukaryotic genomes studied. Increased GC contents at ns sites result in cheaper amino acids, indicating a universal selection for energy efficiency at the start of translation. Conversely, the GC gradients at 4 s and 2 s sites could go in either direction, suggesting different constraints acting at the GC gradients of different codon sites. Within the genome of each species, the GC gradient is intensified from lowly to highly expressed genes. Across species, it also is intensified from metazoan to vertebrata to tetrapoda. We have revealed a conserved selective constraint for cheaper amino acids at the translation start that has resulted in increased GC contents at the ns sites. Elevated GC contents facilitate transcription but result in more stable LSSs around the coding start and subsequently impede translation initiation. This study thus reveals universally increased GC contents at the translation starts of ns sites that are driven by selective constraints in eukaryotic genomes.

## Materials and methods

### Data

cDNA sequences and tabular annotation files in the GFF format for the 259 eukaryotic genomes were downloaded from the Ensembl ftp sites [39]. There are two ftp sites at Ensembl; the main site (ftp.ensembl.org) hosts more frequently used model organisms such as human, mouse, and fruit fly genomes, while the other site (ftp.ensemblgenomes.org) hosts other genomes including fungi, metazoa, plants, and protists. The two sites use different version numbers. We downloaded all available eukaryotic genomes from the release 87 from the main site and the release 33 from the other site. One gene could have multiple cDNAs. In this study, only the one with the longest cDNA was examined.

CDSs and UTR sequences were separated from the downloaded cDNA sequences according to their coordinates in the GFF files using in-house Perl scripts, followed by manual inspection. Unless noted otherwise, a minimal CDS length of 900 bp was required; CDSs shorter than 900 bp were removed from further analyses.

Gene expression data were obtained from the “Baseline experiments” section of Expression Atlas [40], which provides baseline gene expression for different tissues and cell types from selected species.

The amino acid production costs were obtained from Akashi and colleagues [41], and the nucleotide production costs were obtained from Chen and colleagues [7]. These costs refer to *de novo* synthesis costs, *i.e.*, the energetic costs of these information molecules when synthesized from scratch. Detailed information is available in Table S1. Eukaryotic genomes investigated in this study, broad taxonomic groups of their species, and genomic and coding GC contents are listed in Table S2.

### Separating CDSs into different fragments and codon sites

Three fragments, including the starting, middle, and end fragments, were taken from each CDS. Each fragment contained 100 codons (300 bp) in length. Sequences between these fragments, if available, were discarded from further analyses.

Each fragment was initially split into codons, and the three nucleotides in each codon were then classified into 4 s sites, 2 s sites, and ns sites according to the standard codon table.

### Identification of GC gradients in CDSs

Codons were numbered from 1 to 100 according to their positions in the fragments. An average GC content value was calculated for all codons at a certain position across all valid CDSs of a genome. The same was performed for each codon site.

GC gradients were identified by linear regression analysis using an “lm()” function in R, with a formula of “y ∼ x”, where “x” indicates the codon positions ranging from 1 to 100, and ‘y’ indicates the averaged GC contents. A “slope” value would be obtained from the analysis, describing the changes in GC contents per 100 codons; for example, a “slope” value of −5 means that, on average, the GC content decreases 5% for every 100 codons along the direction of translation.

A negative “slope” value indicates decreasing GC content, while a positive “slope” value indicates increasing GC content along the direction of translation.

### Calculation of MFE

RNAfold of the ViennaRNA package [42] was used with default parameters to calculate the MFE of an input sequence.

Unless otherwise noted, the input sequences were 39 bp in length. When the sliding window technique was used, a step size of 3 bp was chosen.

## Competing interests

The authors declare no competing interests.

### CRediT authorship contribution statement

**Na L. Gao:** Methodology, Software, Formal analysis, Visualization, Writing – original draft. **Zilong He:** Methodology, Software, Formal analysis, Writing – original draft. **Qianhui Zhu:** Software, Formal analysis, Visualization. **Puzi Jiang:** Resources, Data curation. **Songnian Hu:** Conceptualization, Supervision, Writing – review & editing. **Wei-Hua Chen:** Conceptualization, Supervision, Writing – review & editing.

## Supplementary material

Supplementary Figure S1 **GC gradients at the start, middle, and end parts of the coding region in selected genomes** X-axis, three coding parts, each span 100 codons.

Supplementary Figure S2 **Statistics on GC gradients at different codon sites and in different gene parts across eukaryotic species** X-axis, GC% change per 100 codons in each of the three gene parts, namely the start (upper panel), middle (middle panel), and end (lower panel) parts. Species are grouped into broad taxonomic groups; the number of species in each group is indicated in the parenthesis next to the group name.

Supplementary Figure S3 **The negative GC gradients at ns sites start from the ∼25^th^ codon and can extend up to the 150th codon** Shown here are averaged GC contents as a function of codon positions, separated by codon sites; left panel: *Homo sapiens*, middle panel: *Danio rerio*, right panel: *Drosophila melanogaster*. Each dot represents the average GC content of the codons at the corresponding codon position across all valid genes of a species (see Methods for details). LOESS lines were plotted to illustrate the general trends. ns, nonsynonymous; 2s, two-fold synonymous; 4s, four-fold synonymous.

Supplementary Figure S4 **Negative GC gradients observed in codons coding for non-essential amino acids and essential amino acids in the human genome** Human essential amino acids include phenylalanine, valine, threonine, tryptophan, methionine, leucine, isoleucine, lysine, and histidine (data obtained from https://en.wikipedia.org/wiki/Essential_amino_acid, accessed on Oct 6, 2018). AAs, amino acids.

Supplementary Figure S5 **Overall GC gradients and gradients at ns sites in selected species after removing codons that code for positively charged amino acids A.** Overall GC contents of the first 100 codons for *Homo sapiens* (red), *Danio rerio* (green), and *Drosophila melanogaster* (blue). Each dot represents the average GC content of the codons at the corresponding codon position across all valid genes of a species (see Methods for details). The linear regression lines were obtained using the 26th to the 100th codons; the first 25 codons were excluded because they were subjected to constraints for less stable LSSs. **B.** Averaged GC contents as a function of codon positions, separated by codon sites. Loess smoothed lines were plotted to illustrate the general trends.

Supplementary Figure S6 **Strengths of GC-gradients at ns site as a function of overall coding GC content** Each point is a genome, color-coded according to the taxonomic groups. Only the 26th to 100th codons were used in the calculation. The strengths of GC gradients were obtained by applying linear regression to each genome and were used to describe the GC content changes along the direction of translation. A negative (positive) GC gradient indicates that the GC content decreases (increases) along the direction of translation.

Supplementary Figure S7 **Strengths of GC gradient of CDSs as a function of overall coding GC content in plant genomes**

Supplementary Table S1 **Costs of *de novo* amino acid and nucleotide synthesis**

Supplementary Table S2 **Eukaryotic genomes used in this study**
