# Supplementary figures and images for: Selection for Cheaper Amino Acids Drives Nucleotide Usage at the Start of Translation in Eukaryotic Genes
Source: Genomics Proteomics Bioinformatics. 2021 Mar 17;19(6):949–57. doi: 10.1016/j.gpb.2021.03.002 (PMC9403032; doi:10.1016/j.gpb.2021.03.002)

*Homo sapiens*

*Danio rerio*

*Drosophila melanogaster*

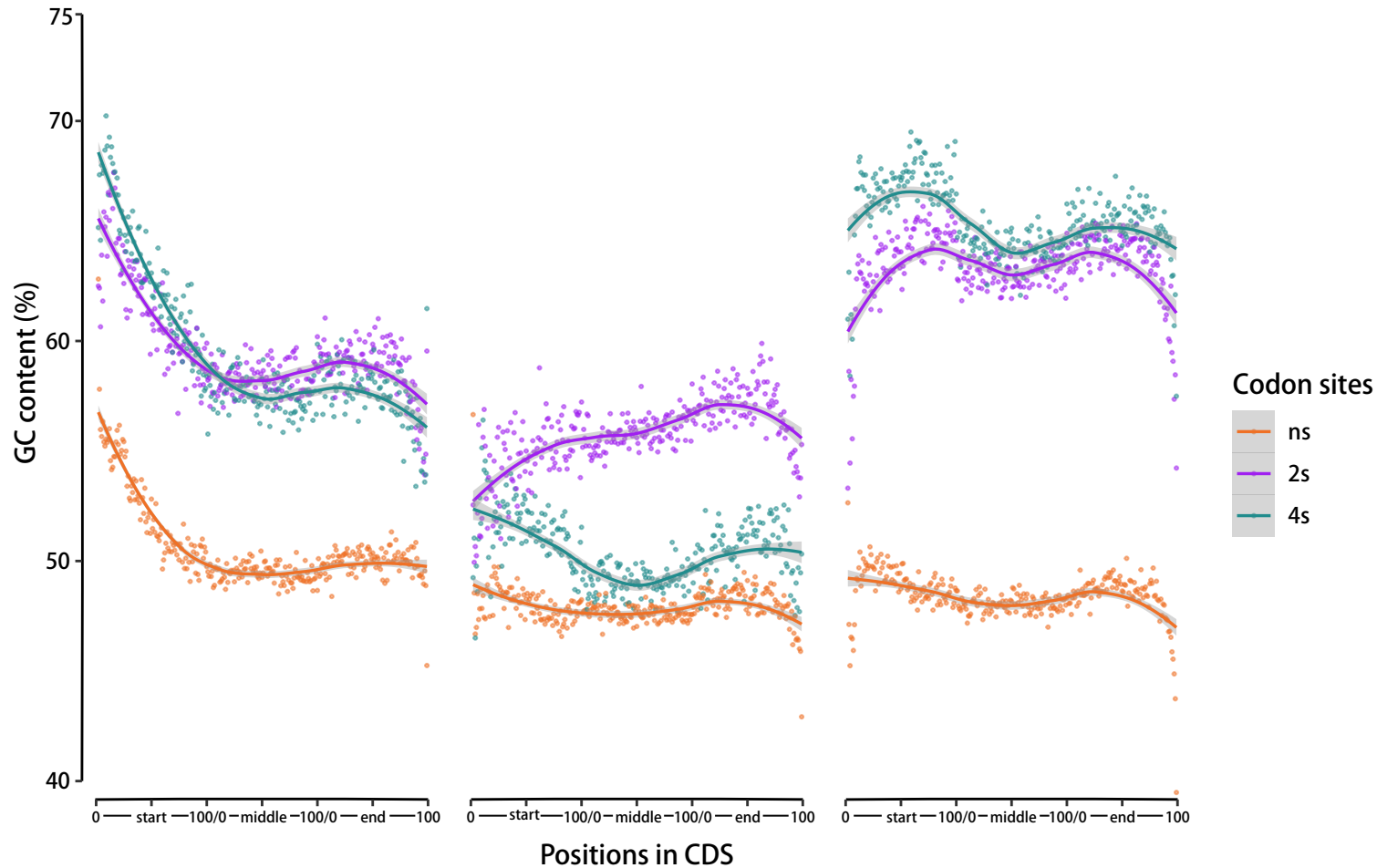

Supplement: Supplementary Figure S1 — GC gradients at the start, middle, and end parts of the coding region in selected genomes X-axis, three coding parts, each span 100 codons. [file mmc1.pdf]

## A Start parts of genes

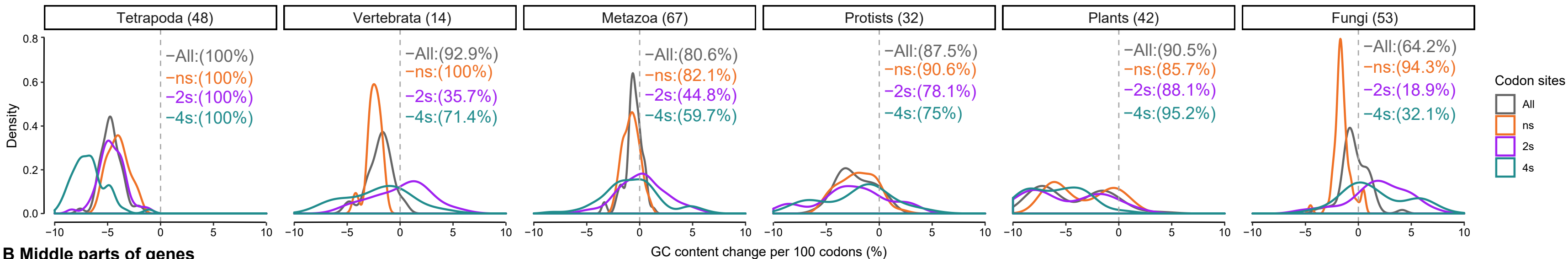

## B Middle parts of genes

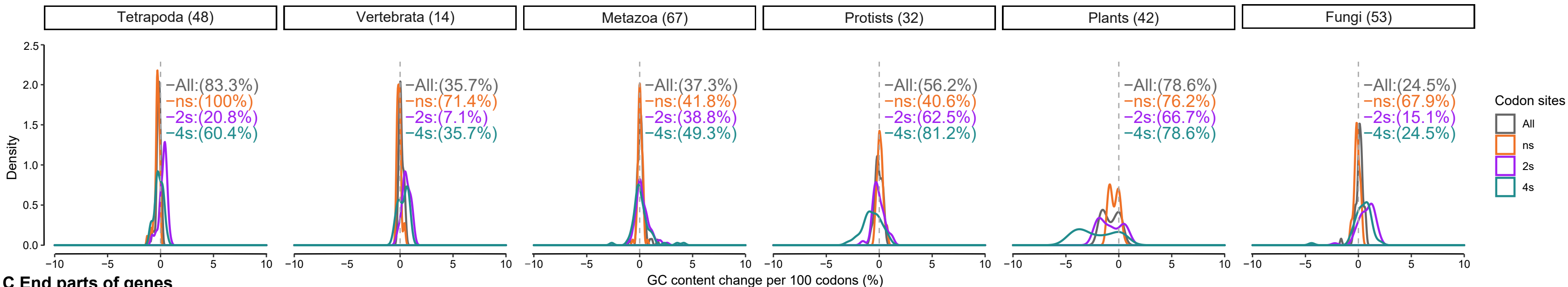

## C End parts of genes

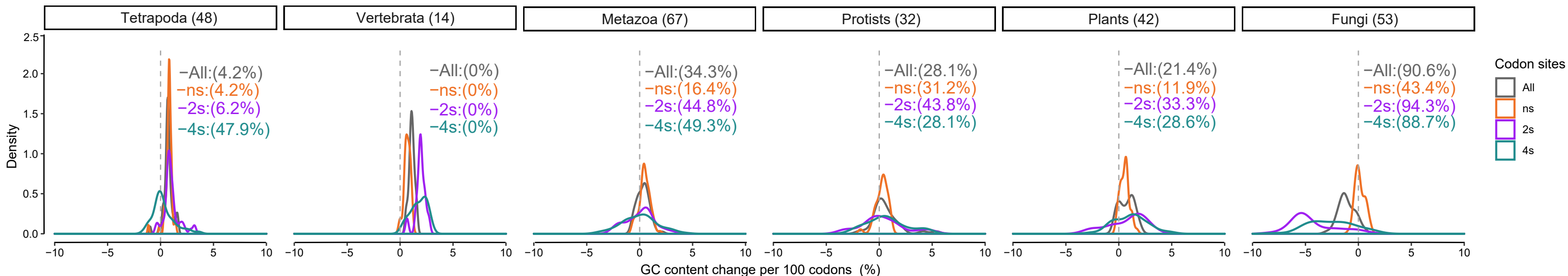

Supplement: Supplementary Figure S2 — Statistics on GC gradients at different codon sites and in different gene parts across eukaryotic species X-axis, GC% change per 100 codons in each of the three gene parts, namely the start (upper panel), middle (middle panel), and end (lower panel) parts. Species are grouped into broad taxonomic groups; the number of species in each group is indicated in the parenthesis next to the group name. [file mmc2.pdf]

*Homo sapiens*

*Danio rerio*

*Drosophila melanogaster*

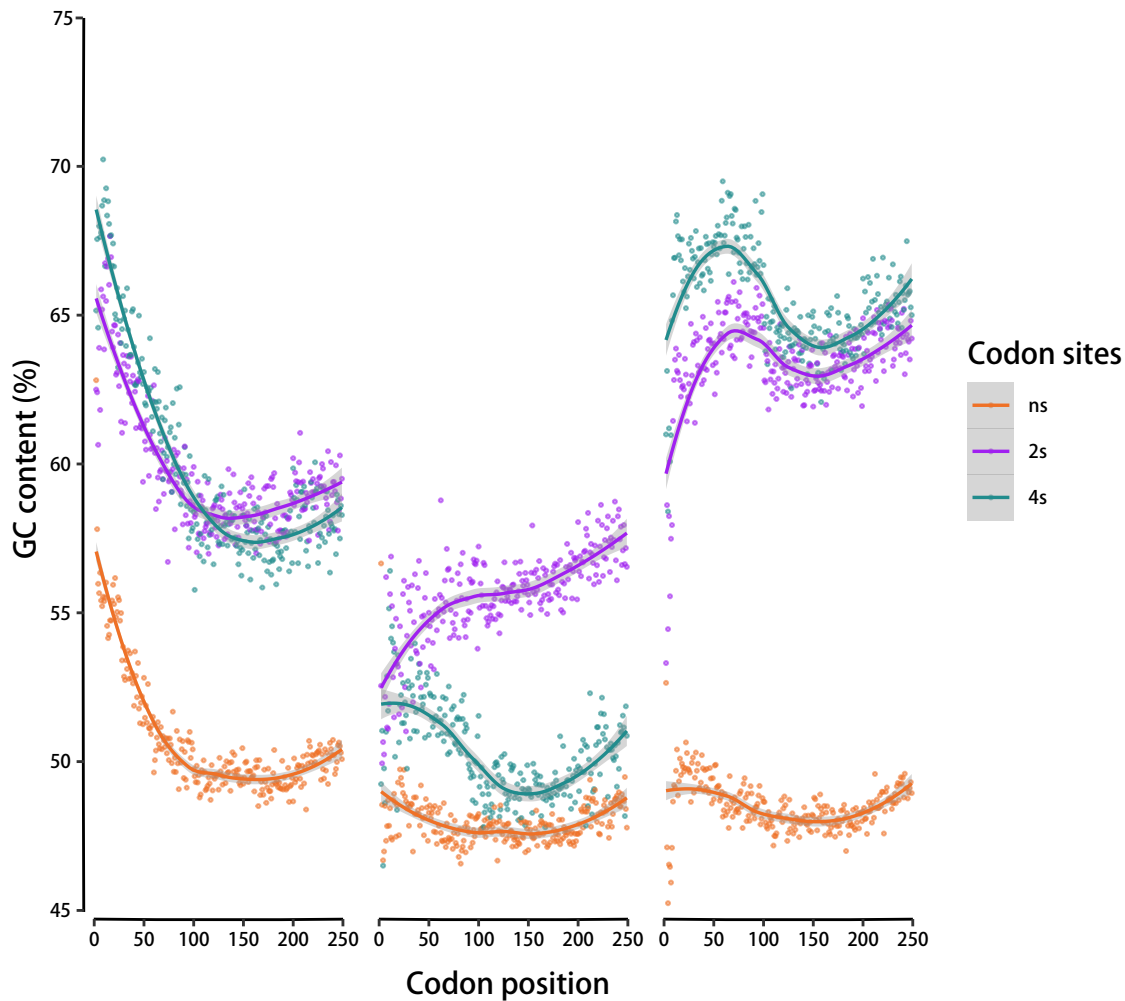

Supplement: Supplementary Figure S3 — The negative GC gradients at ns sites start from the ∼25th codon and can extend up to the 150th codon Shown here are averaged GC contents as a function of codon positions, separated by codon sites; left panel: Homo sapiens, middle panel: Danio rerio, right panel: Drosophila melanogaster. Each dot represents the average GC content of the codons at the corresponding codon position across all valid genes of a species (see Methods for details). LOESS lines were plotted to illustrate the general trends. ns, nonsynonymous; 2s, two-fold synonymous; 4s, four-fold synonymous. [file mmc3.pdf]

Non-essential AAs

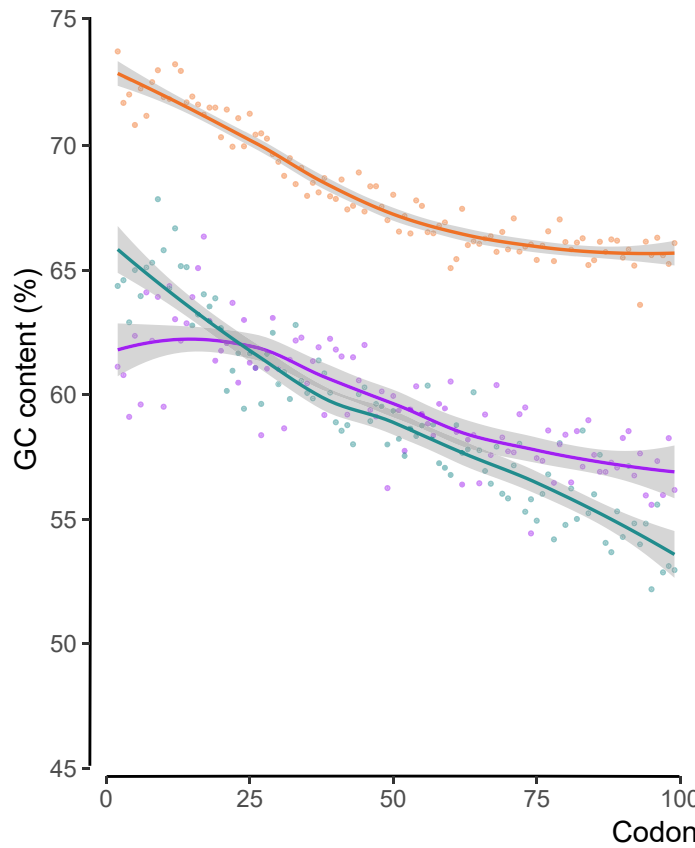

Essential AAs

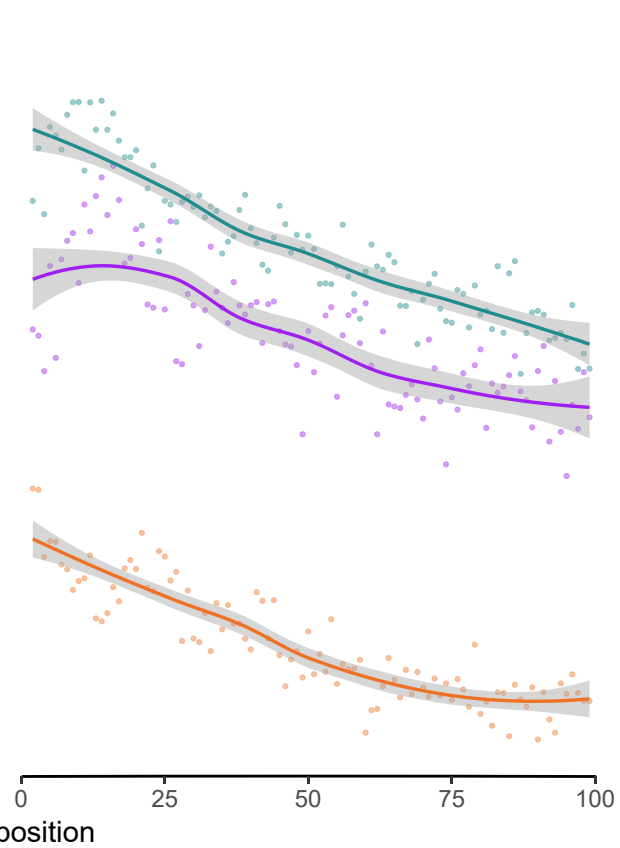

Supplement: Supplementary Figure S4 — Negative GC gradients observed in codons coding for non-essential amino acids and essential amino acids in the human genome Human essential amino acids include phenylalanine, valine, threonine, tryptophan, methionine, leucine, isoleucine, lysine, and histidine (data obtained from https://en.wikipedia.org/wiki/Essential_amino_acid, accessed on Oct 6, 2018). AAs, amino acids. [file mmc4.pdf]

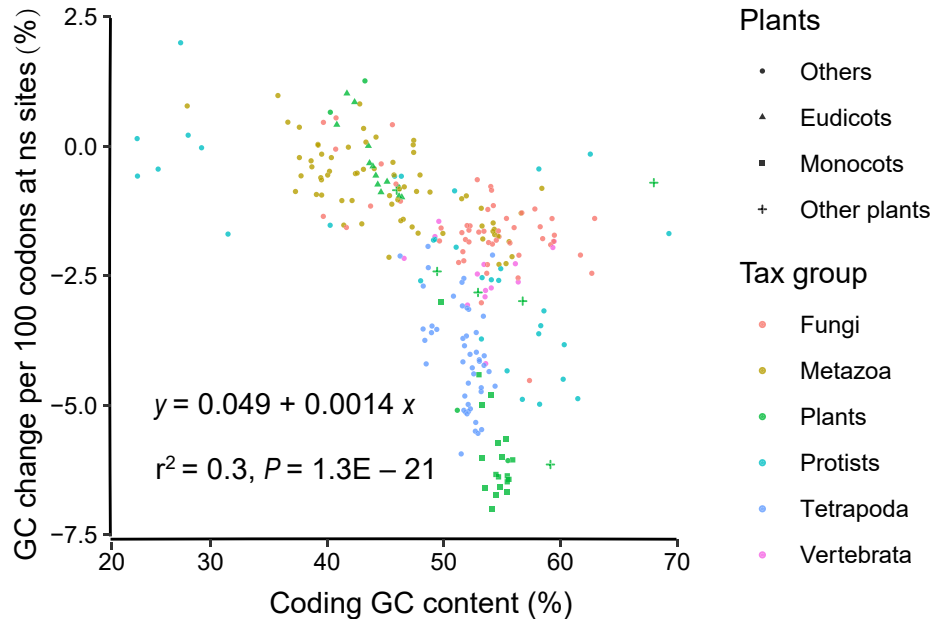

Supplement: Supplementary Figure S5 — Overall GC gradients and gradients at ns sites in selected species after removing codons that code for positively charged amino acids A. Overall GC contents of the first 100 codons for Homo sapiens (red), Danio rerio (green), and Drosophila melanogaster (blue). Each dot represents the average GC content of the codons at the corresponding codon position across all valid genes of a species (see Methods for details). The linear regression lines were obtained using the 26th to the 100th codons; the first 25 codons were excluded because they were subjected to constraints for less stable LSSs. B. Averaged GC contents as a function of codon positions, separated by codon sites. Loess smoothed lines were plotted to illustrate the general trends. [file mmc5.pdf]

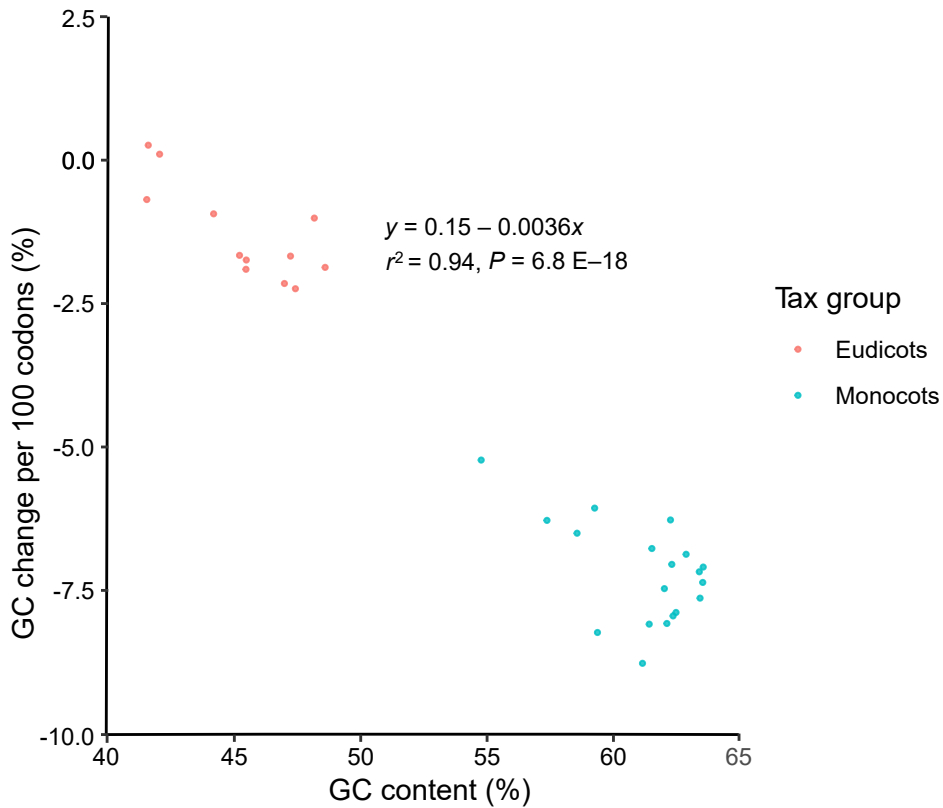

Supplement: Supplementary Figure S6 — Strengths of GC-gradients at ns site as a function of overall coding GC content Each point is a genome, color-coded according to the taxonomic groups. Only the 26th to 100th codons were used in the calculation. The strengths of GC gradients were obtained by applying linear regression to each genome and were used to describe the GC content changes along the direction of translation. A negative (positive) GC gradient indicates that the GC content decreases (increases) along the direction of translation. [file mmc6.pdf]

# *Mus musculus*

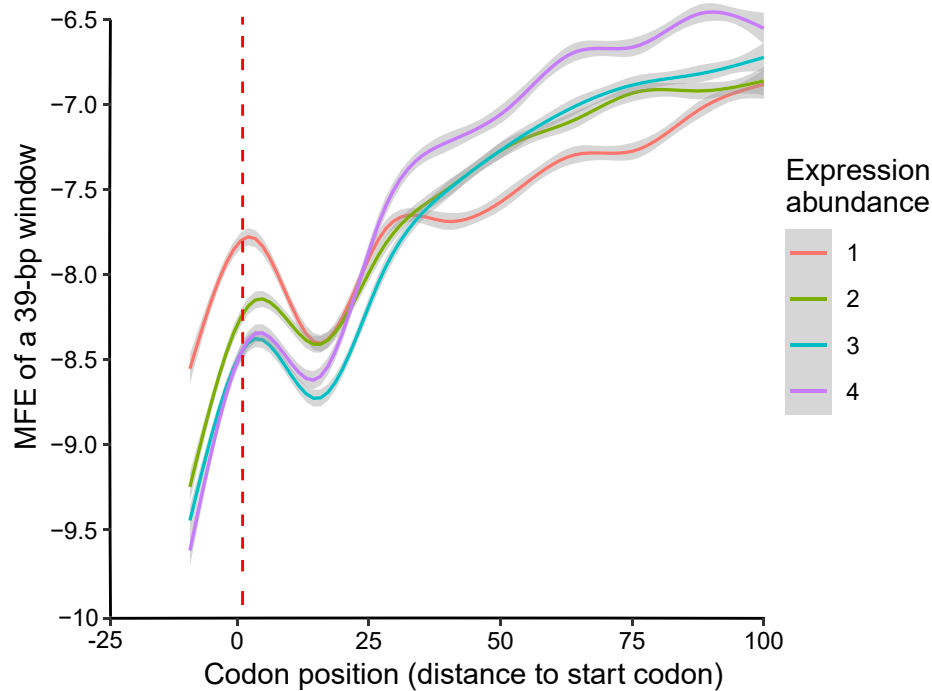

Supplement: Supplementary Figure S7 — Strengths of GC gradient of CDSs as a function of overall coding GC content in plant genomes [file mmc7.pdf]
